# The Burden of Influenza-Associated Hospitalizations in Oman, January 2008-June 2013

**DOI:** 10.1371/journal.pone.0144186

**Published:** 2015-12-07

**Authors:** Salah Al-Awaidy, Sarah Hamid, Idris Al Obaidani, Said Al Baqlani, Suleiman Al Busaidi, Shyam Bawikar, Waleed El-Shoubary, Erica L. Dueger, Mayar M. Said, Emdeldin Elamin, Parag Shah, Maha Talaat

**Affiliations:** 1 Ministry of Health, Muscat, The Sultanate of Oman; 2 Global Disease Detection and Response Program, US Naval Medical Research Unit No.3, Cairo, Egypt; 3 Global Disease Detection Center, Centers for Disease Control and Prevention, Cairo, Egypt; 4 Centers for Disease Control and Prevention, Atlanta, Georgia, United States of America; 5 US Naval Medical Research Unit No.3, Cairo, Egypt; University Hospital San Giovanni Battista di Torino, ITALY

## Abstract

**Introduction:**

Acute respiratory infections (ARI), including influenza, comprise a leading cause of morbidity and mortality worldwide. Influenza surveillance provides important information to inform policy on influenza control and vaccination. While the epidemiology of influenza has been well characterized in western countries, few data exist on influenza epidemiology in the Eastern Mediterranean Region. We describe the epidemiology of influenza virus in Oman.

**Methods:**

Using syndromic case definitions and protocols, patients from four regional hospitals in Oman were enrolled in a descriptive prospective study to characterize the burden of severe acute respiratory infections (SARI) and influenza. Eligible patients provided demographic information as well as oropharyngeal (OP) and nasopharyngeal (NP) swabs. Specimens were tested for influenza A and influenza B; influenza A viruses were subtyped using RT-PCR.

**Results:**

From January 2008 through June 2013, a total of 5,147 cases were enrolled and tested for influenza. Influenza strains were detected in 8% of cases for whom samples were available. Annual incidence rates ranged from 0.5 to 15.4 cases of influenza-associated SARI per 100,000 population. The median age of influenza patients was 6 years with children 0–2 years accounting for 34% of all influenza-associated hospitalizations. By contrast, the median age of non-influenza SARI cases was 1 year with children 0–2 years comprising 59% of SARI. Compared to non-influenza SARI cases, a greater proportion of influenza cases had pre-existing chronic conditions and underwent ventilation during hospitalization.

**Conclusions:**

Influenza virus is associated with a substantial proportion of SARI in Oman. Influenza in Oman approximately follows northern hemisphere seasonality, with major peaks in October to December and a lesser peak around April. The burden of influenza was greatest in children and the elderly. Future efforts should examine the burden of influenza in other potential risk groups such as pregnant women to inform interventions including targeted vaccination.

## Introduction

Acute respiratory infections (ARI) are a major cause of morbidly and mortality. Influenza virus infection is a significant contributor to the causes of ARI with estimates of the annual mortality associated with influenza ranging from 444,200–553,800 deaths globally [1]. In many parts of the world, particularly in temperate regions of the Northern Hemisphere such as the United States and Europe, influenza epidemiology and seasonality have been well characterized. Limited studies describing the epidemiology, seasonality, and burden of seasonal influenza exist in tropical and subtropical regions and in the Eastern Mediterranean region.

In order to improve surveillance and characterize the etiologies and epidemiology of severe acute respiratory infections (SARI) in the region, the Eastern Mediterranean Acute Respiratory Infection Surveillance (EMARIS) network was established. The EMARIS network was initiated through collaboration between the U.S. Centers for Disease Control and Prevention (CDC), U.S. Naval Medical Research Unit No. 3 (NAMRU-3), the World Health Organization (WHO) Eastern Mediterranean Regional Office (EMRO), and the ministries of health of Egypt, Jordan, Oman, and Qatar.

The Sultanate of Oman has a population of 2.8 million. The country has an area of 309.5 thousand square kilometers and is located in the southeastern corner of the Arabian Peninsula with a hot dry interior, humid coastal strip, and a mountainous southern region with seasonal (May to September) monsoon rainfall [2].

The existing literature on influenza in Oman has focused on the demographic and clinical characteristics of patients rather than on influenza seasonality and epidemiology [3–7]. This report describes the establishment of a SARI surveillance system in Oman and presents the epidemiology and seasonality of influenza during the period of January 2008 to June 2013.

## Methods

### Setting and Study Design

Sentinel surveillance for SARI was implemented in four governorate hospitals in Oman in a phased approach. Hospitals were chosen based on covering different geographic regions as well as both urban and rural populations. The initial sentinel site, Sohar Hospital, located in the north of Oman with a catchment population of approximately 343,707 people, began surveillance in January 2008. Ibra Hospital, located in the west of the country with a catchment population of approximately 258,275, joined in September 2008. Sultan Qaboos Hospital, in the south of the country with a catchment population of approximately 369,625, initiated surveillance in December 2009. Nahdha Hospital, a national referral hospital in the capital, Muscat with a population of approximately 1,155,861 initiated surveillance in February 2010.

At each site, staff was trained in surveillance methods, including case definitions, screening, obtaining consent, interviewing, sample collection and reporting. Surveillance staff reviewed hospital admission data on a daily basis and assessed cases with respiratory disease for eligibility. A standardized case interview form was completed for all patients meeting the SARI case definition and for whom consent was obtained. Data collected included demographics, history of current illness, contributing past medical history, as well as information on hospital management and outcome at hospital discharge.

The study used a syndromic case definition standardized across all countries participating in the EMARIS network. SARI case definitions were reviewed annually and modified based on changes in WHO guidance and input from partners. Thus, case definitions used in this study evolved over time. In January, 2008 the WHO SARI case definition (2006) was used for all cases ≥ 5 years of age [8] and for those ≥ 31 days of age and < 5 years, the integrated management of childhood illness (IMCI) pneumonia case definition was used [9]. In January 2010, the project case definition for SARI was modified to encompass all patients ≥31 days of age meeting the CDC International Emerging Infection Program (IEIP) pneumonia case definition [10]. In January 2012, the project SARI case definition was changed to the revised WHO SARI case definition (2011), which applied to all cases of any age [11]. While changes in case definitions were designed to enhance their clarity and ease of use, case definitions adopted in January 2010 and January 2012 were broader, increasing sensitivity (Table 1). SARI cases testing positive for influenza virus were considered influenza-associated hospitalizations.

**Table 1:** Definition and time period of three severe acute respiratory infection (SARI) case definitions used in sentinel surveillance in Oman, January 2008-June 2013.

	Case Definition 1	Case Definition 2	Case Definition 3
**Time Period**	January 2008-December 2009	January 2010-December 2011	January 2012-June 2013
	2–59 months old	≥31 days old	
	+ Hospitalized	+ Hospitalized	
	+ Cough OR tachypnea	+ History of fever	
	+ At least 1 danger sign of pneumonia^a^	OR current fever (≥38°C) OR current hypothermia (<35.5°C)	Hospitalized
		+ At least 1 sign of respiratory infection^b^	+ Cough AND fever (≥38°C) in the last 7 days
	OR	OR	
	≥5 years old	≥31 days old	OR
	+ Hospitalized	+ Hospitalized	Hospitalized
	+ Fever (≥38°C)	+ At least 1 physician assessment criterion^c^	+ Clinically suspected respiratory infection
	+ Cough OR sore throat		
	+ Shortness of breath OR difficulty breathing		

^a^Danger signs of pneumonia included nasal flaring, chest in-drawing, inability to breast-feed, vomiting, grunting, convulsions, stridor, tachypnea, and lethargy.

^b^Signs of respiratory infection included abnormal breath sounds, tachypnea, cough, sputum production, hemoptysis, chest pain, sore throat, and dyspnea.

^c^Physician assessment criteria included severe influenza-like illness, pandemic H1N1 2009, suspected or x-ray confirmed pneumonia, and other respiratory illness.

### Sample Collection and Laboratory Procedures

A physician or nurse obtained both a nasopharyngeal (NP) and oropharyngeal (OP) swab from all patients meeting the SARI case definition. Dacron® swabs were used for specimen collection and both NP/OP swabs were placed in the same 15 ml tube containing 2 ml of viral transport media (VTM). Subsequently, tubes were vigorously agitated for 10 seconds using a vortex mixer. Both swabs were removed and discarded, and the resulting supernatant was decanted into two sterile cryovials labeled with the case’s study ID number and placed immediately in a liquid nitrogen tank to be transported to the Central Public Health Laboratory (CPHL) of the Ministry of Health for viral characterization.

The CPHL tested all samples using real-time RT-PCR, following standard protocols developed by the Centers for Disease Control and Prevention (CDC) in Atlanta, Georgia, USA [12]. Specimens were first tested for influenza A and B virus. Influenza A positive samples were subsequently subtyped. The sample results were sent to the reporting hospital and the Oman Ministry of Health’s Department of Communicable Disease Surveillance and Control (DCDSC). Until 2012, aliquots of samples were sent to the US Naval Medical Research Unit (NAMRU-3) laboratory in Cairo for duplicate testing and quality assurance.

### Data Collection and Management

Data from the standard interview form was entered into a database using EpiData software (Epidata Association, Odense, Denmark). Data from each sentinel site was exported and transmitted to the department of communicable diseases (DCDSC) of the Ministry of Health on a weekly basis. The CPHL maintained an independent database of laboratory results in a Microsoft Excel spreadsheet. Updated electronic versions of this data set were sent to the DCDSC and NAMRU-3 on a weekly basis.

### Statistical Analysis

Demographic and clinical characteristics of the influenza and non-influenza SARI cases were analyzed by calculating frequencies and percentages. Bivariate analysis was performed using χ^2^ tests to compare demographic and clinical characteristics of influenza and non-influenza SARI cases. To assess the role of influenza as a cause of SARI, we calculated the monthly positivity as the proportion of specimens positive for influenza out of the total number of specimens tested each month. The average number of influenza cases identified in a given calendar month across the entire period was calculated. The season was then defined as from the start of the month with the lowest average number of cases to the end of the previous month in the next year (July 1 to June 30). The peak of influenza activity, during a season, was defined as the month with the highest proportion of SARI cases positive for influenza.

Age-stratified annual incidence rates for influenza-associated SARI were estimated using 2013 mid-year population statistics for the governorates served by each hospital [13] (Table 2). Incidence rates were calculated for those seasons in which a full season of surveillance was conducted. As Sohar, Ibra, and Sultan Qaboos hospitals are the only facilities serving North Batina, North Sharqiya, and Dhofar respectively; the populations of these governorates were considered the catchment populations. The catchment population of Nahdha Hospital, a national referral hospital, was challenging to define; thus incidence rates were not estimated for this facility. Statistical analyses were conducted using SAS software version 9.2 (Cary, NC).

**Table 2:** Incidence rates (per 100,000 persons) and 95% confidence intervals for influenza-associated SARI by influenza season and sentinel site.

Season	2008–2009	2009–2010		2010–2011			2011–2012			2012–2013		
Hospital	Sohar	Sohar	Ibra	Sohar	Ibra	SQH	Sohar	Ibra	SQH	Sohar	Ibra	SQH
**0–4 years**	14.5	40.9	32.1	48.3	50.4	81.5	29	22.9	40.7	20.9	36.7	3.7
** **	(7.5–27.8)	(35.0–70.9)	(15.3–67.3)	(33.7–69.0)	(27.9–91.1)	(53.6–123.7)	(18.2–46.0)	(9.5–55.1)	(22.6–73.5)	(12.1–36.0)	(18.3–73.4)	(0.5–26.3)
**5–14 years**	0	24.3	9.9	8.1	3.3	16.3	3.5	3.3	5.4	3.5	3.3	0
** **		(15.9–37.3)	(3.2–30.8)	(3.9–17.0)	(0.5–23.5)	(7.3–36.3)	(1.1–10.8)	(0.5–23.5)	(1.4–21.7)	(1.1–10.8)	(0.5–23.5)	
**15–49 years**	0.9	0.9	1.2	3.7	8.7	5.9	0.2	1.9	0.7	3.5	2.5	0
** **	(0.3–2.5)	(0.3–2.5)	(0.3–5.0)	(2.3–6.1)	(5.1–14.7)	(3.6–9.7)	(0.0–1.7)	(0.6–5.8)	(0.2–3.0)	(2.1–5.8)	(0.9–6.6)	
**50–64 years**	9.6	2.4	7.1	16.8	28.4	14.3	7.2	7.1	3.6	21.5	14.2	3.6
** **	(3.6–25.5)	(0.3–17.0)	(1.0–50.5)	(8.0–35.1)	(10.7–75.7)	(5.4–38.2)	(2.3–22.3)	(1.0–50.5)	(0.5–25.4)	(11.2–41.4)	(3.6–56.8)	(0.5–25.4)
**≥ 65 years**	5.4	5.4	0	21.4	92.2	24	21.4	0	12	48.2	15.4	0
** **	(0.8–38.0)	(0.8–38.0)		(8.0–57.0)	(41.4–205.1)	(6.0–96.0)	(8.0–57.0)		(1.7–85.2)	(25.1–92.6)	(2.2–109.0)	
**All ages**	**2.8**	**9.1**	**5.56**	**10**	**15.4**	**13.5**	**4.5**	**4.3**	**4.6**	**7.7**	**6.8**	**0.5**
** **	**(1.8–4.5)**	**(7.0–11.7)**	**(3.2–9.6)**	**(7.8–12.8)**	**(11.1–21.3)**	**(10.3–17.8)**	**(3.2–6.5)**	**(2.3–7.9)**	**(2.9–7.4)**	**(5.8–10.2)**	**(4.2–11.2)**	**(0.1–2.2)**

### Ethical Considerations

The protocol of the surveillance study was reviewed and approved by the NAMRU-3 Institutional Review Board (IRB), the U.S. CDC IRB, and the National Ethical Review Committee of Oman. As surveillance is considered part of the routine, accepted patient care protocols of hospitals, no written informed consent was required. The IRB approved verbal patient consent, which was documented by placing a study label on the surveillance information sheet, one copy of which was given to the patient and another placed in the patient's medical file. In 2010, SARI surveillance was integrated into Oman’s national influenza and communicable disease surveillance strategy and waiver of consenting procedures was granted.

## Results

From 1 January 2008 through 30 June, 2013, 5,466 patients meeting a SARI case definition were enrolled, of which 94% (5,147/5,466) were tested for influenza virus (Table 3). Among the SARI cases tested, 8% (423/5,147) were positive for influenza.

**Table 3:** Demographic and clinical characteristics of patients enrolled in sentinel surveillance for severe acute respiratory infections by viral influenza etiology, Sultanate of Oman, January 2008-June 2013.

Samples tested for influenza (n = 5147)			
Characteristic	Influenza Positive	Influenza Negative	P Value^a^
(N = Total Known)	(N = 423)	(N = 4724)	
	N (% column total)		
**Age group (N = 5147)**			
**<2 Years**	144 (34)	2781 (59)	<0.01
**2–4 Years**	54 (13)	622 (13)	
**5–14 Years**	53 (13)	335 (7)	
**15–49 Years**	91 (22)	470 (10)	
**50–64 Years**	42 (10)	242 (5)	
**≥65 Years**	39 (9)	274 (6)	
**Male Sex (N = 5138)**	201 (48)	2296 (49)	0.75
**Preexisting Chronic Conditions** ^b^ **(N = 5113)**	150 (35)	1339 (28)	<0.01
**Antibiotic use before admission** ^c^ **(N = 4028)**	68 (16)	684 (14)	0.05
**Antibiotic Use During Hospitalization (N = 5117)**	304 (72)	3477 (74)	0.57
**Intensive Care Unit Admission (N = 4210)**	32 (8)	369 (8)	0.70
**Ventilated (N = 4211)**	18 (4)	121 (3)	0.01
**Died (N = 4618)**	14 (3)	131 (3)	0.52

^a^P-values are based on the chi-square test.

^b^ Chronic conditions include preexisting respiratory, cardiac, renal, hepatic, hematologic, neurologic, infectious, endocrine, and gastrointestinal conditions.

^c^ Antibiotic use in the three days prior to hospital admission.

Incidence estimates for influenza-associated SARI varied by influenza season and health care facility (Table 2). Annual incidence rates ranged from 0.5 to 15.4 cases per 100,000 population. Across all facilities, on average the 0–4 year age group had the highest incidence of influenza-associated SARI (range 32–42 cases per 100,000 population) followed by those aged 65 years and over (12–27 cases per 100,000 population).

Influenza-associated hospitalizations ranged in age from < one month to 87 years of age, with a median age of 6 years (IQR 1–38 years). The median time from illness onset to hospital admission was three days (IQR 2–5) and the median length of stay was also three days (IQR 2–5 days). Preexisting chronic medical conditions were reported by 35% of influenza-associated hospitalizations; the most common condition reported among influenza cases was cardiac disease (10%). Of influenza-associated hospitalizations, 8% required admission to the intensive care unit (Table 3). A higher proportion of those with influenza (4%) required intubation as compared to only 3% of those SARI cases who were influenza negative (p = 0.01).

Over the surveillance period, 14 influenza-associated cases died; six deaths were in cases under five years of age and nine were among females. Chronic medical conditions were reported in 36% (5/14) of those who died compared to 44% (57/129) in non-influenza associated deaths (p = 0.54). Three of the influenza-associated cases who died were reported as admitted to the intensive care unit (ICU) and one was reported as ventilated.

Among influenza-associated cases, 65% (273/423) were positive for influenza A and 36% (151/423) were positive for influenza B. Among Influenza A cases 4% were A(H1N1), 19% were A(H3N2), and 61% were A(H1N1)pdm09; subtyping results were not available for 46 influenza A cases. The median age of A(H1N1)pdm09 cases was 8.5 years (IQR: 1–35) compared to 24 years (IQR: 1–63, p = 0.047) for other influenza A cases and 4 years (IQR: 1–40) for all influenza types (p = 0.44). A smaller proportion (31%) of A(H1N1)pdm09 cases had underlying medical conditions compared to other influenza A cases (47%) (p = .01).

**Fig 1:**
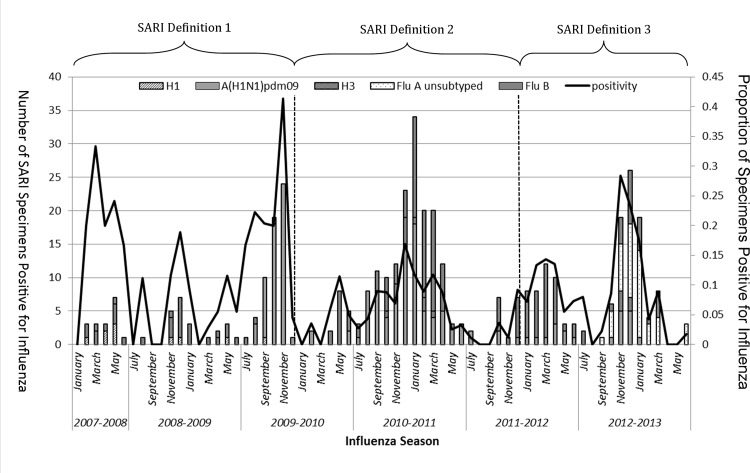
Number of patients enrolled in sentinel surveillance for severe acute respiratory infections by month, Sultanate of Oman, January 2008-June 2013.

On average the month with the lowest number of influenza cases over this study was July. Based on this, data were aggregated and plotted from July 1^st^ of one year through June 30^th^ of the following year. The peak of influenza activity varied across seasons (Fig 1). Influenza activity peaked in November-December in four of five seasons (2008–2009, 2009–2010, 2010–2011, and 2012–2013), while during 2011–2012 season, the only season in which Influenza virus B predominated, influenza activity peaked in March. During the same period globally, influenza activity was at high levels in several other northern hemisphere countries with increasing co-circulation of influenza B virus reported [14]. During the peak of influenza activity of 2009–2010 influenza season, when pandemic influenza A(H1N1)pdm09 predominated, 41% of all SARI cases were due to influenza while in the other seasons the peak of activity ranged from 14% to 19% of SARI cases being positive for influenza (Fig 1). The predominant influenza virus was A(H3N2) (65% of all influenza-positive specimens) in the 2008–2009 season, A(H1N1)pdm09 in the 2009–2010 (78%) and 2010–2011 (51%) seasons, influenza B (67%) in the 2011–2012 season, and unsubtyped influenza A (52%) in the 2012–2013 season.

## Discussion

This report contains the first description of the epidemiology of influenza in Oman covering a period of five years. Overall 8% of SARI cases were influenza-associated and during peaks of influenza activity 14% to 41% of SARI was influenza-associated. Influenza-associated hospitalizations were significantly older than non-influenza SARI cases. While influenza-associated deaths were uncommon (3% of influenza associated hospitalizations), deaths among SARI cases were significantly more common among females and those who reported pre-existing chronic medical conditions. An annual seasonal pattern of influenza activity was noted with the nadir occurring during the hottest season consistent with northern hemisphere seasonality. Based on influenza A subtyping alone, the predominant virus in each season matched influenza A subtypes included in the northern hemisphere influenza vaccine available that season.

Influenza surveillance in Oman appeared to provide data consistent with those reported by similar systems elsewhere. Influenza positivity among SARI cases (8%) was comparable to that reported in Asia and Africa (8–11%)[15–19] but lower than the 27% positivity reported in a recent study of influenza-associated SARI in Eastern Europe [20]. The system proved sensitive enough to detect the emergence of the 2009 influenza pandemic. Furthermore, in non-pandemic years, the predominant influenza viruses detected were consistent with those reported elsewhere in the northern hemisphere [21].

There are a number of potential limitations to this study. Isolation of influenza virus from a patient meeting the SARI case definition does not prove causality as influenza has been isolated from asymptomatic individuals in several studies [22]. Although the SARI case definition broadened over the course of the study, it likely does not fully capture all cases that might be hospitalized with respiratory illness due to influenza [11]. Moreover, it is likely that the broadening of the case definition resulted in increases in the number of patients enrolled and influenza cases detected. There may also have been limitations in the performance of surveillance. All participating hospitals provided care across the age spectrum, but SARI cases were predominately under five years of age and there were few deaths among the elderly. It is possible that the SARI case definition is less sensitive for elderly patients than for pediatric cases, that there was less surveillance among medical wards with elderly patients, or that healthcare seeking behavior of the elderly differs from that of children with a greater tendency for health seeking in young children. While efforts were made to regularly train staff in active case finding, no audits were done to assess how well or how completely the case definitions were applied. Consequently, there is the potential that differential effort was made in capturing pediatric versus adult cases for enrollment and testing.

The higher incidence of influenza-associated SARI in the 2010–11 season also bears explanation. As aforementioned, no audits were done to assess the completeness of case ascertainment; however, it is possible that the detection of the pandemic influenza A(H1N1)pdm09 in July 2009 led to heightened awareness about influenza and greater adherence to surveillance protocols. This hypothesis is supported by WHO Global Influenza Surveillance and Response System (GISRS) data, which show an increase in positive influenza specimens beginning in 2010, predominantly of the subtype influenza A(H1N1)pdm09.[23]

The exclusion of Nahdha Hospital, a large referral hospital located in the capital, Muscat, may have impacted findings. The catchment population of Nahdha Hospital overlaps with three other tertiary care and two other secondary care hospitals that receive SARI cases in Muscat and more severe cases may go to two of these other facilities (the Royal and University hospitals). Therefore, estimates from Nahdha Hospital would most probably underestimate the true SARI incidence. Determining the denominator population for use in the incidence estimate of this large referral hospital would have been problematic hence Nahdha Hosptial was excluded from analyses.

While there is potential bias in this system, the epidemiology of influenza shown in our data is consistent with that seen in other northern hemisphere temperate countries including the emergence of the 2009 pandemic. At the same time, the pooling of data across the time of the emergence of the pandemic may introduce bias in our data. The epidemiology of pandemic influenza has been documented to be different from that of seasonal H1N1 and H3N2 viruses with a higher burden among school-age children and young adults.[24]

Initiating influenza surveillance in Oman has had a number of important benefits. Firstly, it provides important information to drive policy on influenza control in Oman. Since 2001, the Oman Ministry of Heath has recommended and offered influenza vaccine to healthcare workers at no cost. The collection of high quality surveillance data provides an opportunity to begin to establish the burden of influenza and may begin to identify other risk-groups that might be targeted for vaccination.

Another important benefit of establishing influenza surveillance is that it builds laboratory capacity to detect a range of pathogens using molecular diagnostic techniques. This improved capacity adds to the global ability to detect novel influenza viruses such as H5N1 and H7N9 and creates a platform for the detection of other non-influenza respiratory viral pathogens, such as MERS-CoV, which are emerging threats to Oman and the gulf region [25].

Pregnancy has been associated with increased risk for adverse outcomes related to influenza [26]. While there was an attempt to capture pregnancy status, the number of pregnancies reported in this study was limited. Though the overall mortality was low, we saw a higher proportion of deaths among females. This could have been due to differences in care seeking behavior or due to disproportionate risk factors for poor outcome from influenza, such as pregnancy, that were not adequately captured in the surveillance data.

This study provides a first look at the epidemiology and burden of influenza across the Sultanate of Oman. Additional work is needed to better assess the burden of influenza including incidence rates adjusted for health care seeking behavior, and to characterize which groups are disproportionally affected. In addition, consideration should be made to assessing the financial burden on the government and its people. The Ministry of Health has already begun a program of vaccination of healthcare workers. It is hoped that this study will lead to better data to drive vaccination policy in other segments of the population.

## References

[1] Lozano R, Naghavi M, Foreman K, Lim S, Shibuya K, Aboyans V, et al Global and regional mortality from 235 causes of death for 20 age groups in 1990 and 2010: a systematic analysis for the Global Burden of Disease Study 2010. Lancet 2012 12 15;380(9859):2095–2128. 10.1016/S0140-6736(12)61728-0 23245604 PMC10790329

[2] Central Intelligence Agency. The World Factbook. Available at: https://www.cia.gov/library/publications/the-world-factbook/geos/mu.html. Accessed 16 May 2014.

[3] Al-Mahrezi A, Samir N, Al-Zakwani I, Al-Muharmi Z, Balkhair A, Al-Shafaee M. Clinical characteristics of influenza A H1N1 versus other influenza-like illnesses amongst outpatients attending a university health center in Oman. Int J Infect Dis 2012 7;16(7):e504–7. 10.1016/j.ijid.2012.02.015 22521779

[4] Pajankar S, Al Qassabi SS, Al Harthi SM. Clinical Features and Outcome of 65 Laboratory Confirmed Cases of H1N1 in Muscat, Sultanate of Oman. Oman Med J 2012 5;27(3):201–206. 10.5001/omj.2012.46 PMC3394368 22811768

[5] Al-Lawati J, Al-Tamtami N, Al-Qasmi A, Al-Jardani A, Al-Abri S, Al Busaidy S. Hospitalised patients with Influenza A (H1N1) in the Royal Hospital, Oman: Experience of a tertiary care hospital, July-December 2009. Sultan Qaboos Univ Med J 2010 12;10(3):326–334. 21509252 PMC3074727

[6] Ahmad AS, Puttaswamy C, Mudasser S, Abdelaziz O. Clinical Presentation and Outcome in Hospitalized Patients of 2009 Pandemic Influenza A (H1N1) viral infection in Oman. Oman Med J 2011 9;26(5):329–336. 10.5001/omj.2011.82 22125727 PMC3215438

[7] Al-Muharrmi Z. Understanding the Influenza A H1N1 2009 Pandemic. Sultan Qaboos Univ Med J 2010 8;10(2):187–195. 21509228 PMC3074714

[8] World Health Organization. PAHO-CDC Generic Protocol for Influenza Surveillance. Available at: http://www.paho.org/English/AD/DPC/CD/flu-snl-gpis.htm. Accessed 11 October 2013.

[9] World Health Organization. Handbook IMCI. Integrated management of childhood illness. 2005.

[10] International Emerging Infections Program—Thailand. Active Surveillance for Pneumonia Requiring Hospitalization in Rural Thailand. Available at: https://www.ieip.in.th/info/TSSEARS.asp. Accessed 26 May 2014.

[11] World Health Organization. WHO global technical consultation: global standards and tools for influenza surveillance. 2011.

[12] Templeton KE, Scheltinga SA, Beersma MF, Kroes AC, Claas EC. Rapid and sensitive method using multiplex real-time PCR for diagnosis of infections by influenza a and influenza B viruses, respiratory syncytial virus, and parainfluenza viruses 1, 2, 3, and 4. J Clin Microbiol 2004 4;42(4):1564–1569. 15071005 10.1128/JCM.42.4.1564-1569.2004 PMC387552

[13] National Centre for Statistics and Information. Available at: http://www.ncsi.gov.om/Pages/NCSI.aspx. Accessed 15 June 2015.

[14] World Health Organization. Influenza Virus Activity in the World, 13 April 2012. Available at: http://www.who.int/influenza/gisrs_laboratory/updates/summaryreport_20120413/en/. Accessed 15 February 2015.

[15] Azziz-Baumgartner E, Alamgir AS, Rahman M, Homaira N, Sohel BM, Sharker MA, et al Incidence of influenza-like illness and severe acute respiratory infection during three influenza seasons in Bangladesh, 2008–2010. Bull World Health Organ 2012 1 1;90(1):12–19. 10.2471/BLT.11.090209 22271960 PMC3260571

[16] Radin JM, Katz MA, Tempia S, Talla Nzussouo N, Davis R, Duque J, et al Influenza surveillance in 15 countries in Africa, 2006–2010. J Infect Dis 2012 12 15;206 Suppl 1:S14–21. 10.1093/infdis/jis606 23169960

[17] Tallo VL, Kamigaki T, Tan AG, Pamaran RR, Alday PP, Mercado ES, et al Estimating influenza outpatients' and inpatients' incidences from 2009 to 2011 in a tropical urban setting in the Philippines. Influenza Other Respir Viruses 2014 3;8(2):159–168. 10.1111/irv.12223 24393336 PMC4186463

[18] Muyembe Tamfum JJ, Nkwembe E, Bi Shamamba SK, Bankoshi F, Ilunga BK, Katz KA, et al Sentinel surveillance for influenza-like illness, severe acute respiratory illness, and laboratory-confirmed influenza in Kinshasa, Democratic Republic of Congo, 2009–2011. J Infect Dis 2012 12 15;206 Suppl 1:S36–40. 10.1093/infdis/jis537 23169969

[19] Emukule GO, Khagayi S, McMorrow ML, Ochola R, Otieno N, Widdowson MA, et al The burden of influenza and RSV among inpatients and outpatients in rural western Kenya, 2009–2012. PLoS One 2014 8 18;9(8):e105543 10.1371/journal.pone.0105543 25133576 PMC4136876

[20] Meerhoff TJ, Simaku A, Ulqinaku D, Torosyan L, Gribkova N, Shimanovich V, et al Surveillance for severe acute respiratory infections (SARI) in hospitals in the WHO European region—an exploratory analysis of risk factors for a severe outcome in influenza-positive SARI cases. BMC Infect Dis 2015 1 8;15:1-014-0722-x. 10.1186/s12879-014-0722-x PMC4314771 25567701

[21] World Health Organization. Influenza virological updates. 2015; Available at: http://www.who.int/influenza/gisrs_laboratory/updates/en/. Accessed 15 February 2015.

[22] Patrozou E, Mermel LA. Does influenza transmission occur from asymptomatic infection or prior to symptom onset? Public Health Rep 2009 Mar-Apr;124(2):193–196. 19320359 10.1177/003335490912400205 PMC2646474

[23] World Health Organization. FluNET—CHARTS. 2015; Available at: http://www.who.int/influenza/gisrs_laboratory/flunet/charts/en/. Accessed 21 October 2015.

[24] Karageorgopoulos DE, Vouloumanou EK, Korbila IP, Kapaskelis A, Falagas ME. Age distribution of cases of 2009 (H1N1) pandemic influenza in comparison with seasonal influenza. PLoS One 2011;6(7):e21690 10.1371/journal.pone.0021690 21747947 PMC3128617

[25] World Health Organization. Background and summary of novel coronavirus infection–as of 21 December 2012. Available at: http://www.who.int/csr/disease/coronavirus_infections/update_20121221/en/index.html. Accessed 29 June 2014.

[26] Van Kerkhove MD, Vandemaele KA, Shinde V, Jaramillo-Gutierrez G, Koukounari A, Donnelly CA, et al Risk factors for severe outcomes following 2009 influenza A (H1N1) infection: a global pooled analysis. PLoS Med 2011 7;8(7):e1001053 10.1371/journal.pmed.1001053 21750667 PMC3130021

